# Drivers of plateau adaptability in cashmere goats revealed by genomic and transcriptomic analyses

**DOI:** 10.1186/s12864-023-09333-1

**Published:** 2023-08-01

**Authors:** Cuiling Wu, Shengchao Ma, Bingru Zhao, Chongkai Qin, Yujiang Wu, Jiang Di, Langda Suo, Xuefeng Fu

**Affiliations:** 1 Key Laboratory of Special Environments Biodiversity Application and Regulation in Xinjiang, School of Life Sciences, Xinjiang Normal University, Xinjiang, Urumqi, 830017 China; 2 Key Laboratory of Genetics Breeding and Reproduction of Xinjiang Wool-sheep Cashmere-goat (XJYS1105), Institute of Animal Science, Xinjiang Academy of Animal Sciences, Xinjiang, Urumqi, 830011 China; 3 College of Animal Science, Xinjiang Agricultural University, Xinjiang, Urumqi, 830052 China; 4 College of Animal Science and Technology, Nanjing Agricultural University, Nanjing, 210095 Jiangsu China; 5 Xinjiang Aksu Prefecture Animal Husbandry Technology Extension Center, Xinjiang Aksu, 843000 China; 6 Institute of Animal Science, Tibet Academy of Agricultural and Animal Husbandry Sciences, Tibet Lhasa, 850009 China

**Keywords:** Plateau adaptability, Tibetan and jiangnan cashmere goats, Genetic, Gene expression and PSGs

## Abstract

**Background:**

The adaptive evolution of plateau indigenous animals is a current research focus. However, phenotypic adaptation is complex and may involve the interactions between multiple genes or pathways, many of which remain unclear. As a kind of livestock with important economic value, cashmere goat has a high ability of plateau adaptation, which provides us with good materials for studying the molecular regulation mechanism of animal plateau adaptation.

**Results:**

In this study, 32 Jiangnan (J) and 32 Tibetan (T) cashmere goats were sequenced at an average of 10. Phylogenetic, population structure, and linkage disequilibrium analyses showed that natural selection or domestication has resulted in obvious differences in genome structure between the two breeds. Subsequently, 553 J vs. T and 608 T vs. J potential selected genes (PSGs) were screened. These PSGs showed potential relationships with various phenotypes, including myocardial development and activity (*LOC106502520*, *ATP2A2*, *LOC102181869*, *LOC106502520*, *MYL2*, *ISL1*, and *LOC102181869* genes), pigmentation (*MITF* and *KITLG* genes), hair follicles/hair growth (*YAP1*, *POGLUT1*, *AAK1*, *HES1*, *WNT1*, *PRKAA1*, *TNKS*, *WNT5A*, *VAX2*, *RSPO4*, *CSNK1G1, PHLPP2*, *CHRM2*, *PDGFRB*, *PRKAA1*, *MAP2K1*, *IRS1*, *LPAR1*, *PTEN*, *PRLR*, *IBSP*, *CCNE2*, *CHAD*, *ITGB7*, *TEK*, *JAK2*, and *FGF21* genes), and carcinogenesis (*UBE2R2*, *PIGU*, *DIABLO*, *NOL4L*, *STK3*, *MAP4*, *ADGRG1*, *CDC25A*, *DSG3*, *LEPR*, *PRKAA1*, *IKBKB*, and *ABCG2* genes). Phenotypic analysis showed that Tibetan cashmere goats has finer cashmere than Jiangnan cashmere goats, which may allow cashmere goats to better adapt to the cold environment in the Tibetan plateau. Meanwhile, *KRTs* and *KAPs* expression in Jiangnan cashmere goat skin was significantly lower than in Tibetan cashmere goat.

**Conclusions:**

The mutations in these PSGs maybe closely related to the plateau adaptation ability of cashmere goats. In addition, the expression differences of *KRTs* and *KAPs* may directly determine phenotypic differences in cashmere fineness between the two breeds. In conclusion, this study provide a reference for further studying plateau adaptive mechanism in animals and goat breeding.

**Supplementary Information:**

The online version contains supplementary material available at 10.1186/s12864-023-09333-1.

## Background

Changes in altitude gradients on Earth create a complex and changeable natural climate. Meanwhile, ladder-like topographical due to altitude differences is particularly evident in China; Tibet is the region with the highest altitude and the most unique climate in China. The high altitude has led to extreme climate conditions, such as high cold, hypoxia, and strong ultraviolet radiation (UVR). This extreme climate not only challenges animal’s physiological tolerance, but also adversely affects the ecosystems of the Tibet area, further leading to a decrease in the quality and quantity of livestock feed, reduced water availability, and increased disease prevalence [1, 2]. Therefore, extreme climates have limited the development of animal husbandry in Tibet area, which accounts for one-eighth of China’s total area.

To cope with extreme climate, numerous plateau indigenous animals (e.g., *Bos mutus*, *Equus kiang*, *Procapra picticaudata*, and *Tetraogallus tibetanus*, etc.) have developed a unique adaptive mechanism during evolution, specifically reflected at physiological, cellular, and molecular levels, e.g., skin tone deepening [3], a well-developed cardiopulmonary system and a high-density capillary network are formed [4, 5], and increased VEGF expression [6], etc. As such, they are excellent subjects for research on adaptive evolution. Physiological changes in plateau adaptation are often accompanied by genetic changes. Previous studies mainly focused on the former, while studies on the molecular mechanism are relatively rare, particularly in non-model organisms. Previously, research focused on known candidate genes and analyzed the differences in their sequences between animals at high and low altitudes to identify the specific variation in candidate genes of high-altitude animals. This type of research is somewhat limited because it focuses on the optimal potential adaptive phenotype genes, and makes it difficult to find new adaptive mechanisms. For example, research mainly focused on hypoxia adaptation, while ignoring cold or high radiation adaptation. Moreover, the evolution of complex adaptive physiological characteristics often results from the activity of numerous genes, and this method may ignore other important functional genes [7, 8]. Currently, advances in sequencing technologies have permitted extensive comparative genomics studies across species or populations at different altitudes. For example, Qiu et al. sequenced the genome and transcriptome of the female yak and compared them with the cattle reference genome, finding expansion of the sensory perception and energy metabolism related gene families in the yak, and metabolic enzyme and hypoxia adaptation related genes had undergone positive selection [9]. In addition, Ge et al. determined that genes associated to repair, function, angiogenesis, and hypoxia were positively selected in Tibetan antelopes, based on genome construction [10]. Further, Qu et al. performed whole-genome sequencing in ground tits and compared it to other breeds (great and yellow-cheeked tits). In ground tits, the gene family related to energy metabolism had expanded while that related to immunity and smell had contracted and the genes related to hypoxia response and bone development had undergone positive selection [11].

In contrast to the genomic research, transcriptomic analysis reveals genome-wide gene expression characteristics of animal organs, tissues, or cells at different times. If the genome sequence does not produce relevant variation to adapt to extreme climates, gene transcription can also effectively result in adaptation to extreme climates. Therefore, gene expression levels are an important molecular phenotype for identifying key genes responding to physiological challenges [12]. For example, the transcriptomic studies found accelerated evolution of genome-wide gene expression levels in Schizothorax (fish endemic to the Qinghai-Tibet Plateau), particularly in genes related to hypoxia and energy metabolism, compared with zebrafish [13, 14]. In conclusion, integrating genomics and transcriptomics can reveal the regulatory mechanisms of the adaptability of plateau indigenous animals and the development of new breeds resistant to low temperature, hypoxia, and UVR.

Cashmere goats (*Capra hircus*) were domesticated from wild goats (Bezoar, *Capra aegagrus*) in the Fertile Crescent of the Near East about 10,000 years ago, and then got spread to Europe, Africa, Asia through migration and trade, basically. At present, cashmere goat is widely found in Tibet, Xinjiang, and other regions of China. Jiangnan cashmere goat and Tibetan cashmere goat are important breeds in Xinjiang and Tibet, respectively. They were all formed by crossbreeding, and Liaoning cashmere goat as the male parent, local cashmere goat as the female parent. Cashmere goats provide inhabitants with essential living resources such as meat, milk, cashmere, and fur, and are an important income source for local herdsmen. In addition, cashmere goats have stronger adaptability to extreme climates (e.g., high cold, hypoxia, and UVR). Although within the same longitude range, the Xinjiang region is lower in altitude and has a climate more suitable for animal survival than Tibet. Thence, we used 32 Jiangnan and 32 Tibetan cashmere goats as research objects, extracted genomic DNA from their blood, and further performed whole genome resequencing of them. At the same time, we obtained a skin tissue transcriptome dataset of Tibetan and Jiangnan cashmere goats. Based on comparative genomic and transcriptomic analysis between these two breeds, we extensively screened for candidate genes associated with plateau adaptive, further investigating the plateau adaptive expression strategies of related genes (The specific experimental procedure is shown in Figure S1). In conclusion, this study provide a theoretical basis for further study the molecular regulatory mechanism of animal plateau adaptability and goat breeding.

## Results

### Genome resequencing and variation

First, the quality control was performed on the whole genome resequencing data of the 64 cashmere goats. After quality control, the clean data was mapped to the goat reference genome; the mapping rates were > 99%, 1X Coverage > 94%, and 4X Coverage > 91% of all samples. Finally, we performed SNP detection of 64 samples and obtained 15,349,261 high-quality SNPs through filtering and screening. The SNP detection statistics are shown in Table S1.

### Population structure

**Fig. 1:**
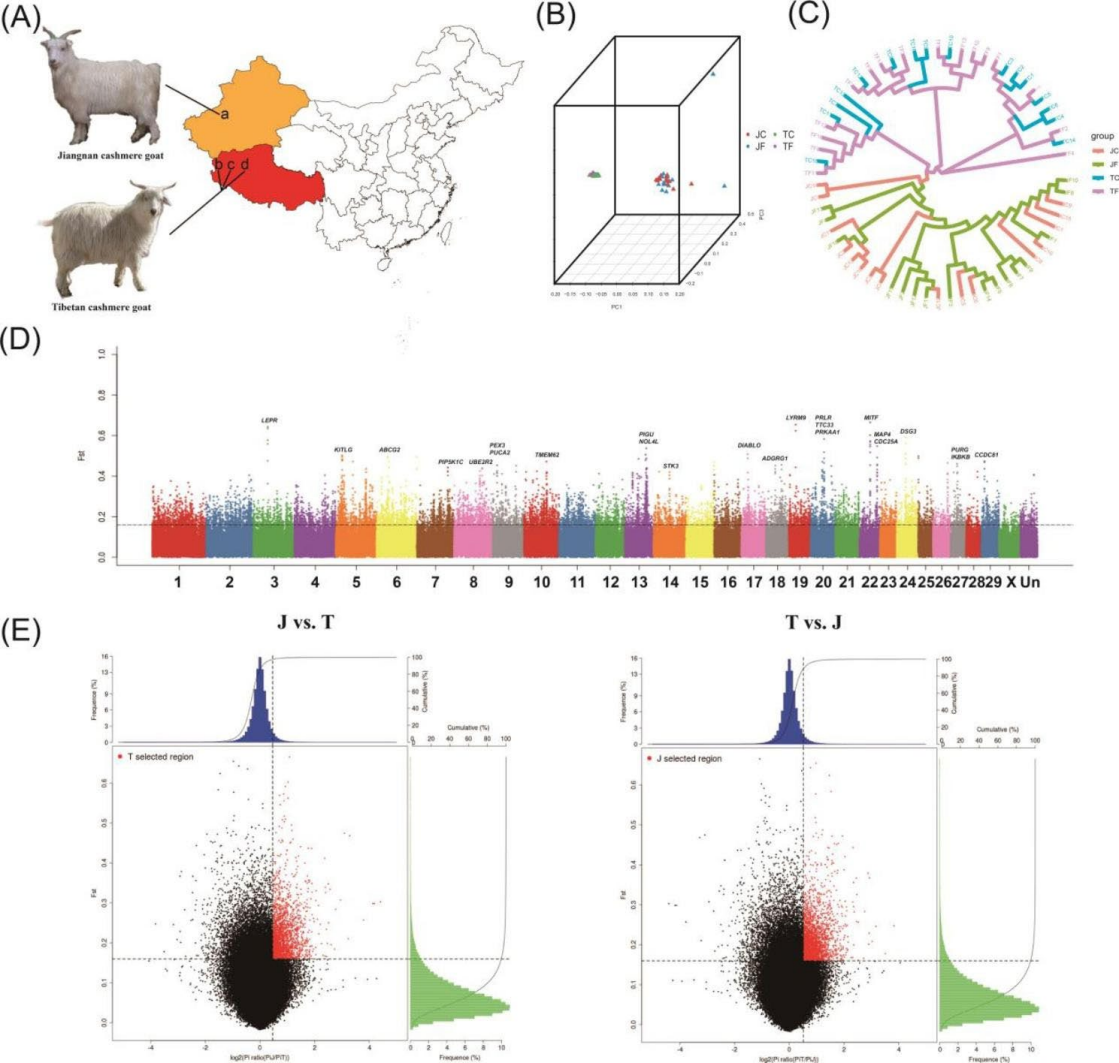
**Sampling and genomic landscape of the divergence of Tibetan and Jiangnan cashmere goat breeds.** (A) Map of cashmere goat sampling. The orange and red areas in the figure are the sampling areas of Jiangnan and Tibetan cashmere goat, respectively. In the map, a is Aksu Prefecture; b, c, and d are Ritu County, Gaize County, and Nima County, respectively. (B) Principal components of cashmere goat samples. (C) Phylogenetic tree of 64 cashmere goats. (D) FST in 5-kb sliding windows across autosomes between Tibetan and Jiangnan cashmere goats. (E) FST Pi selection elimination analysis. The X-axis of the scatter diagram is the log_2_(Pi (π) ratio) value and the Y-axis is the FST value, which correspond to the frequency distribution diagram above the scatter diagram and the frequency distribution diagram on the right, respectively. The area of red scatter points is the top 5% area selected by Pi (π) ratio and FST.

PCA divided Tibetan and Jiangnan cashmere goats into two separate clusters (Fig. 1B). There was a significant correlation between the clustering pattern of the two cashmere goat breeds and the locations where they were collected. Phylogenetic trees showed that both Tibetan and Jiangnan cashmere goats derive from a single domestication event or a common ancestor (Fig. 1C). In addition, admixture analysis (k = 2, 3, and 4) showed no obvious gene flow between Tibetan and Jiangnan cashmere goats due to geographical isolation (Figure S2). Moreover, LD (linkage disequilibrium) decayed more slowly in Tibetan cashmere goats than in Jiangnan cashmere goats (Figure S3).

### Selective sweeps

We scanned the genome for regions with extreme FST divergence and the highest differences in Pi (π) ratio in 5-kb sliding windows in two compare groups (J vs. T and T vs. J) to further detect the PSGs on chromosomes (Fig. 1D F).

In total, we identified 553 J vs. T PSGs (based on π and Fst, selected genes in the top 5% region) and 608 T vs. J PSGs (based on π and Fst, selected genes in the top 5% region). GO and KEGG enrichment analysis of the PSGs in J vs. T showed that they were mainly enriched in cell adhesion (GO:0007155), positive regulation of apoptotic process (GO:0043065), extracellular region (GO:0005576), cytosol (GO:0005829), and centrosome (GO:0005813). Interestingly, *PARP1*, *BMF*, *CDC25A*, and *ATR* are associated with cellular responses to UV (GO:0034644), *CD6* and *ELANE* are associated with acute inflammatory responses to antigenic stimulus (GO:0002438), and *LTA* and *TNF* genes are associated with positive regulation of chronic inflammatory responses to antigenic stimulus (GO:0002876) (Table S2 and S3).

GO and KEGG analysis in T vs. J further showed that PSGs were mainly enriched in the following functions: response to nuclear speck (GO:0016607), cytosol (GO:0005829), nucleoplasm (GO:0005654), calcium ion binding (GO:0005509), small GTPase binding (GO:0031267), zinc ion binding (GO:0008270), regulation of actin cytoskeleton (chx04810), and PI3K-Akt signaling pathway (chx04151). Notably, PSGs were enriched for ATP binding (GO:0005524) in J vs. T and T vs. J. Moreover, *LOC106502520*, *ACTN3*, and *LOC102181869* are associated with regulation of skeletal muscle contraction force (GO:0014728); *LOC106502520*, *MYL2*, *ISL1*, and *LOC102181869* genes are associated with ventricular cardiac muscle tissue morphogenesis (GO:0055010); *LOC106502520*, *ATP2A2*, and *LOC102181869* are associated with regulation of heart contraction force (GO:0002026), cardiac muscle hypertrophy in response to stress (GO:0014898), or regulation of slow-twitch skeletal muscle fiber contraction (GO:0031449); *PRKAA1*, *TNKS*, *WNT5A*, *VAX2*, *RSPO4*, and *CSNK1G1* are associated with Wnt signaling (GO:0016055); *DUOX1*, *WRN*, *PNKP*, *GCLM*, and *DUOX2* are associated with response to oxidative stress (GO:0006979); and *PRKAA1*, *LOC106502520*, *MYL2*, *DAG1*, *ATP2A2*, *ITGB7*, and *LOC102181869* are associated with hypertrophic cardiomyopathy (chx05410) (Table S2 and S3).

### Gene expression profiles in skin tissues of Jiangnan and Tibetan cashmere goats

**Fig. 2:**
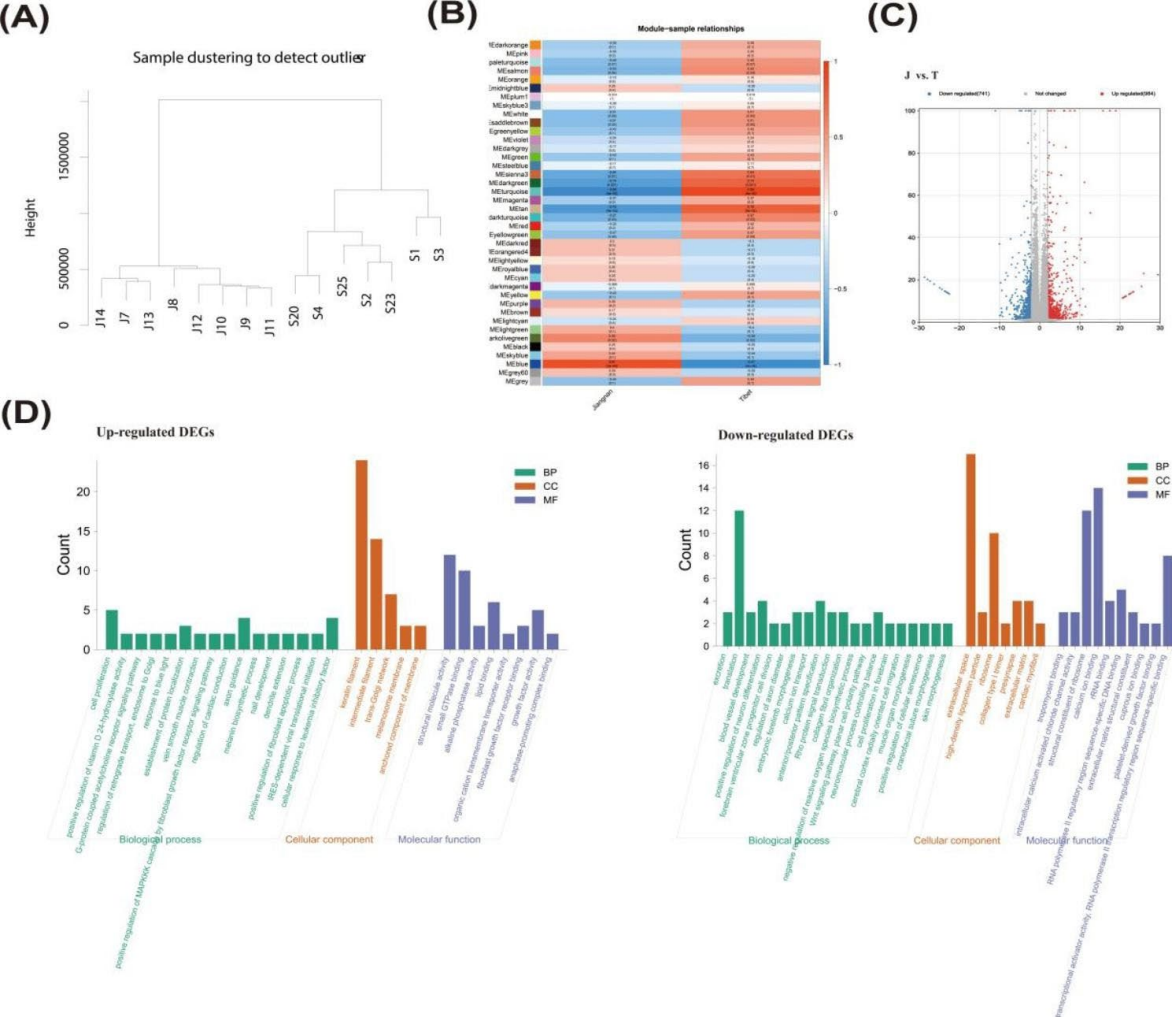
**transcriptomic analysis of Jiangnan and Tibetan cashmere goat skin tissue.** (A) Clustering tree of 15 Jiangnan or Tibetan cashmere goat skin transcriptomes. (B) Expression patterns of genes within different color modules in skin tissues of Tibetan and Jiangnan cashmere goats. The values in the rectangles represent the correlation between gene expression patterns in different modules and breeds. (C) Volcano plot of DEGs. (D) GO enrichment analysis of DEGs.

Genome-wide gene expression pattern cluster analysis showed that, eight Tibetan cashmere goats and seven Jiangnan cashmere goats were clustered into different clusters, respectively (Fig. 2A). However, Ce individuals and Fe individuals are not fully distinguished in the cluster of eight Tibetan cashmere goats or seven Jiangnan cashmere goats. WGCNA analysis further assigned genome-wide genes into modules of different colors, and the results showed that all modules showed distinct differential expression patterns in Tibetan and Jiangnan cashmere goats (Fig. 2B).

The analysis of DEGs showed that 1426 genes (log_2_FC ≥ 2 or ≤ − 2, p < 0.01) were differentially expressed in Tibetan and Jiangnan cashmere goats, of which 653 DEGs were up-regulated and 773 DEGs were down-regulated (Fig. 2C). GO and KEGG enrichment of DEGs showed that (Fig. 2 and Table S4), the up-regulated DEGs were predominantly enriched into keratin filament (GO:0045095), intermediate filament (GO:0005882), structural molecule activity (GO:0005198), and small GTPase binding (GO:0031267). Furthermore, it is also worth noting that, *EDNRB* and *EDN2* genes are enriched for vein smooth muscle contraction (GO:0014826), *FGF23* and *FGF21* genes are enriched for positive regulation of MAPKKK cascade by fibroblast growth factor receptor signaling pathway (GO:0090080), *LOC102170378* and *AGT* genes are enriched for regulation of cardiac conduction (GO:1,903,779), *GPR143*, *DCT*, and *TYR* genes are enriched for melanin biosynthetic process (GO:0042438) and melanosome membrane (GO:0033162), etc. Then, the down-regulated DEGs were predominantly enriched into translation (GO:0006412), extracellular space (GO:0005615), ribosome (GO:0005840), structural constituent of ribosome (GO:0003735), and calcium ion binding (GO:0005509). It is still worth noting that, *WNT7A* and *CTHRC1* genes are enriched for Wnt signaling pathway, planar cell polarity pathway (GO:0060071), *MYH7B* and *TNNT2* genes are enriched for cardiac myofibril (GO:0097512), etc.

### The candidate genes associated with cashmere growth in Jiangnan and Tibetan cashmere goats

**Fig. 3:**
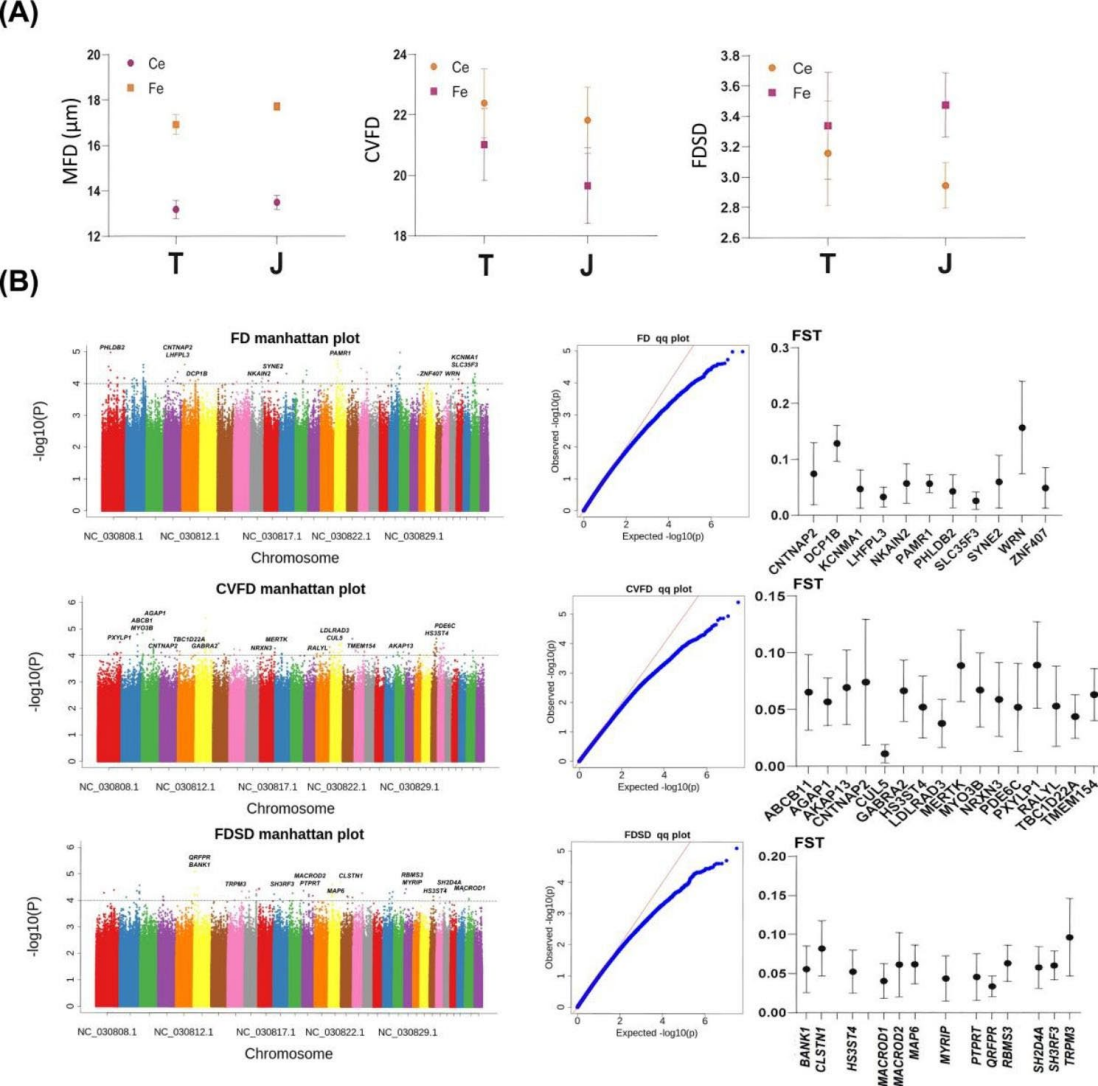
**SNPs or candidate genes associated with cashmere fineness.** (A) Statistics of three cashmere fineness phenotypes in Tibetan and Jiangnan cashmere goats. (B) GWAS of cashmere fineness, and selective sweeps analysis of trait-associated candidate genes

We measured the cashmere MFD of the 64 goats (Tibetan and Jiangnan cashmere goats) in this study, and further calculated the CVFD and FDSD (Fig. 3A). Meanwhile, we found that the MFD of Jiangnan cashmere goats is higher than that of Tibetan cashmere goats. All phenotypic data were used for GWAS.

GWAS further showed that some SNPs were associated with the MFD, FDSD, and CVFD of Tibetan or Jiangnan cashmere goats. These SNPs were annotated to 11 (MFD), 16 (FDSD), and 13 (CVFD) genes, respectively (Fig. 3B). Combined with selective sweeps, these genes were found in regions with low genetic diversity (Fig. 3B). This may indicate that cashmere fineness has a similar genetic basis in different goat breeds. We further focused on the expression patterns of candidate genes in Tibetan and Jiangnan cashmere goats, including *SLC35F3* (Down), *PAMR1* (Down), *DCP1B* (Up), *HS3ST4* (Down), *AKAP13* (Down), *MYO3B* (Down), *GABRA2* (Up), *RALYL* (Up), *QRFPR* (Down), *TRPM3* (Down), *PTPRT* (Up), *MAP6* (Up), and *MACROD2* (Up); they all had significant differential expression (log_2_FC ≥ 1 or ≤ − 1, *P* < 0.05;Figure S4). Simultaneously, down-regulated DEG *HS3ST4* was associated with both FDSD and CVFD. However, there is no evidence showing that these candidate genes regulate wool growth and follicle development in animals. Hence, the effect of these genes on cashmere fineness remains to be verified.

In addition, keratin (KRTs) and keratin-associated proteins (KAPs) are the main structural proteins of animal hair and play a decisive role in its physical properties [15, 16]. In transcriptomic analysis, 24 differentially expressed *KRT* or *KAP* genes (including *LOC102178483*, *KRT82*, *LOC102179595*, *LOC102185436*, *LOC10218376*, *LOC102184223*, *LOC102183211*, *LOC108638297*, *LOC102184693*, *LOC102185150*, *LOC102170546*, *LOC102177517*, *KRT84*, *LOC100861184*, *LOC108638292*, *LOC102172755*, *LOC102171368*, *LOC102170264*, *LOC108638299*, *LOC108638288*, *LOC102176522*, *LOC108634945*, *LOC102174594*, and *LOC102182538*) were enriched in keratin filaments (GO:0045095), and 14 differentially expressed *KRT* or *KAP* genes (Including *LOC102179881*, *KRTAP11-1*, *KRTAP15-1*, *KRT35*, *LOC100861175*, *LOC100861181*, *LOC100861381*, *LOC102176726*, *LOC100860930*, *LOC102168573*, *KRT39*, *KRT26*, *LOC102176457*, and *KAP8*) were enriched in intermediate filaments (GO:0005882). The expression of the above genes in the skin tissue of Tibetan cashmere goats was significantly lower than that of Jiangnan cashmere goats (log_2_FC of the above genes >2). This may explain the finer texture of Tibetan cashmere.

### The PSGs associated with pigmentation in Jiangnan and Tibetan cashmere goats

**Fig. 4:**
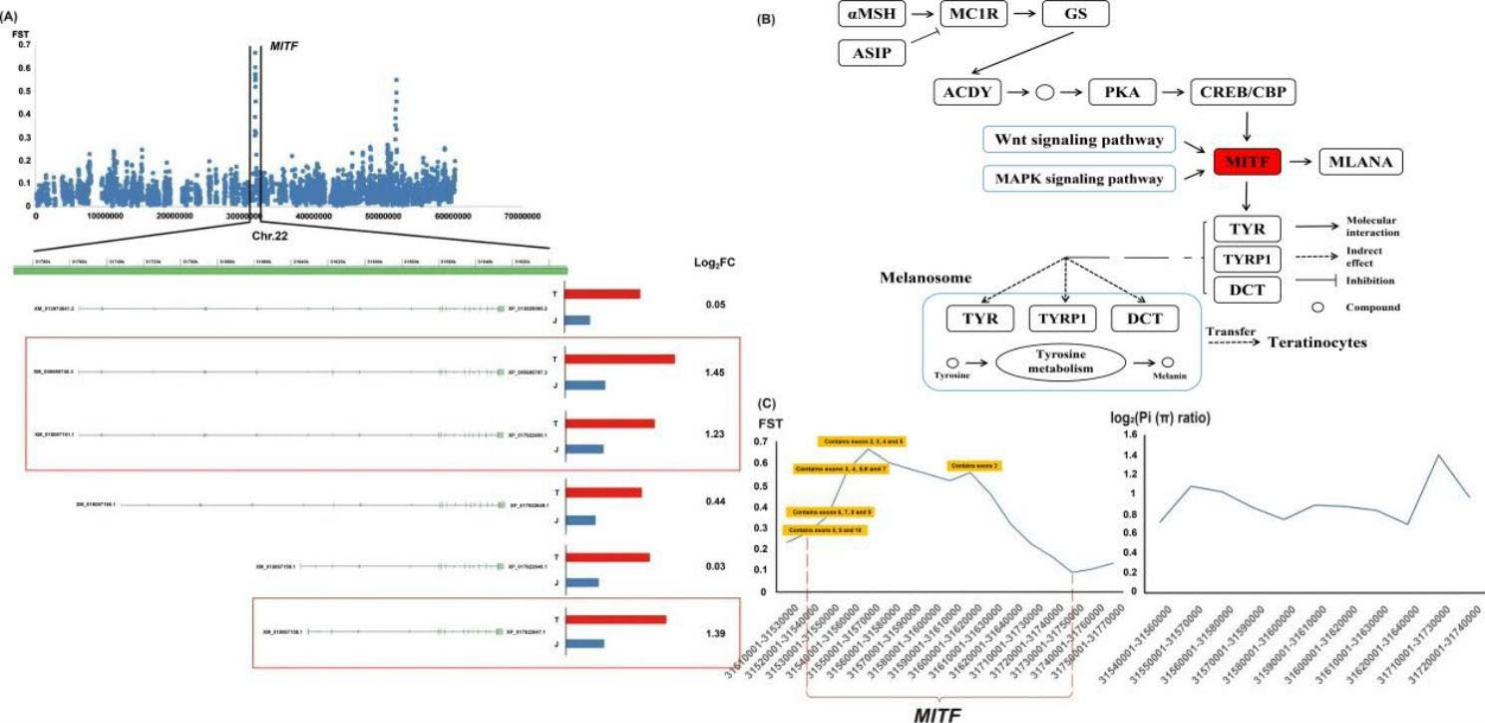
**Functional genomic basis of differences in melanin synthesis between Tibetan and Jiangnan cashmere goats.** (A) FST of all SNPs in chromosome 22 between Tibetan and Jiangnan cashmere goats. (B) Schematic overview of the melanogenesis pathway. The schematic was drew based on KEGG imagery [62–64]. (C) FST and Pi (π) ratio of *MITF* gene on chromosome 22

In T vs. J, we found two PSGs–K*ITLG* and microphthalmia-associated transcription factor (*MITF*) genes on chromosome 5 and 22(Figs. 1D and 4A), respectively. *MITF* and *KITLG* genes play a crucial role in the melanin biosynthesis and pigmentation [17, 18]. Among them, MITF plays an important role in mammalian skin and hair melanin deposition, mainly regulating the transcription of three pigmentation enzymes, TYR, TYRP-1, and TYRP-2/DCT (Fig. 4B) [18–21].

In the goat reference genome, we found seven transcripts of *MITF* genes: X1, X2, X3, X4, and X5-1–3. transcriptomic analysis showed high *MITF* expression in the skin of both cashmere goat breads, but the average *MITF* expression level in the skin of Tibetan cashmere goats was higher than in Jiangnan cashmere goats. We further analyzed the expression characteristics of the seven *MITF* transcripts in Jiangnan and Tibetan cashmere goat skin tissue; the expression levels of X2 (XM_005695740.3), X5-1 (XM_018067161.1) and X4 (XM_018067158.1) in Tibetan cashmere goat skin tissue were significantly higher than those in Jiangnan cashmere goats (The log_2_FC of these 3 transcripts > 2)(Fig. 4A). However, the expression levels of the three downstream genes (*TYR*, *TYRP1*, and *DCT*) regulated by *MITF* gene were very low (FPKMs of all three genes: 100 ~ 0). Based on selective sweeps analysis, the sequence segment of *MITF* gene where exons 2, 3, 4, and 5 are located was most obvious selected for environmental adaptation (Fig. 4C). However, *TYR*, *TYRP1* and *DCT* genes were not selected, and their sequence structures may be relatively conserved among different breeds of cashmere goat.

## Discussion

Extreme environments (low temperature, low oxygen, and UVR) have adverse effects on the survival of plateau organisms, limiting the development of animal husbandry in plateau areas. The adaptation of indigenous animals like cashmere goats to the extreme plateau environment can help us understand the molecular regulatory mechanisms underlying their independent adaptation to the plateau environment.

During long-term domestication and genetic improvement of Tibetan and Jiangnan cashmere goats, the blood of Liaoning cashmere goat was introduced to improve their reproductive performance. The Tibetan and Jiangnan cashmere goat populations in this study shared a common ancestor. Phylogenetic and PCA analysis of 64 cashmere goats showed that, even if these two breeds may have originated from the same ancestor, long-term environmental adaptation and selection separated them. LD analysis further revealed that the two breeds were subject to varying degrees of natural or artificial selection. Based on population structure analysis and genome-wide gene expression pattern analysis, the environmental differences (particularly regional differences in altitude) and geographic isolation led to differences in genome sequence structure and genome-wide expression patterns between these two breeds. The differences between the two groups indicate that cashmere goats adapted to the extreme environment of the plateau by changing their genome structure and gene expression levels. Selective sweep analysis further identified a total of 553 J vs. T and 608 T vs. J PSGs associated with environmental adaptation; these PSGs may function in different adaptive mechanisms in response to numerous adverse environment factors.

Among the various physiological activities of animals, respiration is the most important for animal survival. However, hypoxia is one of the most significant features of the Tibetan plateau, which will inevitably affect animal breathing. Based on previous studies, the physiological and morphological characteristics of different animals’ adaptation to hypoxia are different. Some plateau native animals have strong cardiopulmonary functions and dense capillaries, such as yak, plateau bar-headed geese, and Tibetan antelope. Studies have also shown that the plateau pika responds to the cold and hypoxic plateau environment by maintaining a high resting metabolic rate, non-shivering heat production, and improving oxygen utilization [22]. The adaptation of deer mice to the plateau is achieved by increasing the blood oxygen affinity. The studies have shown that increased oxygen affinity of deer mice with the altitude increase and is related to the distribution frequency of alleles at different altitudes [23]. In our study, three PSGs were related to regulation of the heart contraction force (including *LOC106502520*, *ATP2A2*, and *LOC102181869*) and four PSGs related to ventricular cardiac muscle tissue morphogenesis (Including *LOC106502520*, *MYL2*, *ISL1*, and *LOC102181869*) were selected in Tibetan cashmere goats; the mutations in the above genes may enhance the myocardial function of Tibetan cashmere goats.

Another environmental characteristic of the plateau is the cold; the thick hair on the animal’s body helps resisting the cold. Our study found that, compared with Jiangnan cashmere goats, Tibetan cashmere goats have finer cashmere. The mathematical theory of reaction diffusion proposed by Nagorcka et al. [24] considered hair follicle size as ultimate determinant of the volume and fiber diameter of the dermal papilla. Moore’s progenitor cell theory also supports this view [25]. Moreover, Adelson et al. found that fiber diameter and hair follicle density are inversely proportional [26]. Based on the above results, we consider that Tibetan cashmere goats may have smaller and denser hair follicles, which play a positive role in resisting the cold and UVR. In follow-up GWAS, a few SNPs associated with cashmere fineness traits (MFD, CVFD, and FDSD) were identified; the candidate genes obtained by SNP annotation were not directly associated with hair follicle or hair growth. In selective sweep analysis, we found PSGs enriched in positive regulation of Notch signaling pathway (involving the PSGs *YAP1*, *POGLUT1*, *AAK1*, *HES1*, and *WNT1*), Wnt signaling pathway (involving the PSGs *PRKAA1*, *TNKS*, *WNT5A*, *VAX2*, *RSPO4*, and *CSNK1G1*), and PI3K-Akt signaling pathway (Involving the PSGs *PHLPP2*, *CHRM2*, *PDGFRB*, *PRKAA1*, *MAP2K1*, *IRS1*, *LPAR1*, *PTEN*, *PRLR*, *IBSP*, *CCNE2*, *CHAD*, *ITGB7*, *TEK*, *JAK2*, and *FGF21*) were affected by environmental adaptive selection. Demehri et al. [27] found that ligand binding to Notch receptors will activate the hair follicle stem cells, so that the hair follicles enter the growth phase. Andl et al. [28] found that WNT signals are required for the initiation of hair follicle development. Lu et al. [29] found that amphiregulin promotes hair regeneration of skin-derived precursors via PI3K pathways. Therefore, we speculate that a mutation in the above PSGs may regulate differences in hair follicle development between the two cashmere goat breeds, thereby affecting cashmere fineness traits. Meanwhile, we found that the expression levels of *KRT* and *KAP* genes in the skin tissue of Tibetan cashmere goats were significantly lower than those of Jiangnan cashmere goats, which may be another key factor determining cashmere fineness.

The extreme climate increases the risk of cancer in plateau animals, such as gastric or skin cancer [30–32]. Among them, the increase of animal skin cancer caused by the increased solar UVR has attracted global attention; this also poses a serious threat to natural ecology, especially reproduction. The skin is the largest protective organ, and the melanin in it is the main component shielding UVR [33]. Melanin is secreted, generated, and stored by melanocytes, mainly distributed in the epidermis and dermis and subcutis [34, 35], and its quantity is related to biological species, location, age, etc. Melanin prevents skin and organ damage by absorbing UV photons and free radicals [36] to protect DNA from UV damage [37]. In this study, *MITF* and *KITLG* were environmental adaptive and selected; these two genes are closely related to melanin biosynthesis and transport. The expression level of three *MITF* transcripts in the skin tissue of Tibetan cashmere goat was higher than in Jiangnan cashmere goats. Hence, we speculate that sequence mutation due to environmental adaptive selection may be an important factor affecting *MITF* gene expression. However, we noticed that *TYR*, *TYRP1*, and *DCT*, which are directly involved in melanin synthesis had low expression levels, directly regulated by *MITF*. In addition, these three genes were not subjected to strong environmental adaptive selection. Therefore, we speculate that the maturation period (Sampling period) may not be critical for melanin synthesis and deposition in the skin tissues of the two cashmere goat breeds. At the same time, we have no direct evidence of a significant difference in melanin deposition in the skin tissues between the two breeds. Nonetheless, we do not exclude environmental adaptation mutations in the *MITF* and *KITLG* at different growth stages between the two cashmere goat breeds.

In addition to the above-mentioned regulatory mechanisms in response to cancer, we found that PSGs (J vs. T PSGs: *KITLG*, *UBE2R2*, PIGU, *NOL4L*, *STK3, DIABLO*, *ADGRG1*, *MAP4*, *CDC25A*, *DSG3*, and *MITF*) with Pi (π) ratio > 0.5 and FST > 0.4 were more or less associated with cancer (For example: *UBE2R2* gene–cervical cancer [38]; *PIGU* and *DIABLO* genes–colorectal cancer [39, 40]; *NOL4L*, *STK3* and *MAP4* genes–ovarian cancer [41–43]; *ADGRG1* gene–tumorigenesis [44]; *CDC25A* gene–human tumors [45]; *DSG3* gene–head and neck cancer [46]; *LEPR*, *PRKAA1*, and *IKBKB* genes–gastric cancer [47–49]; *ABCG2* gene–breast cancer [50]). GO enrichment analysis also found that five PSGs (including *AREL1*, *TNIP1*, *DUOXA1*, *DUOXA2*, and *JAK2*) associated with inflammatory response conditions were also selected for environmental adaptation. In summary, we speculate that, the environmental adaptive selection in PSGs related to diseases such as cancer is the most obvious.

## Conclusion

In this study, we identified the 553 J vs. T and 608 T vs. J PSGs in cashmere goats, which are mainly associated with carcinogenesis, pigmentation, myocardial tissue development, and hair growth. Meanwhile, the specific functions of these PSGs still deserve further study. In addition, we found that the plateau adaptation of cashmere goats was also closely related to the genome-wide gene expression pattern. For example, compared with the Jiangnan cashmere goat, the cashmere of the Tibetan cashmere goat is significantly finer, which may be because the expression levels of *KRT* and *KAP* gene family members in the skin tissue of the Jiangnan cashmere goat were significantly lower than that of the Tibetan cashmere goat.

## Materials and methods

### Experimental animals

A total of 32 1-year-old female Jiangnan cashmere goats were selected from the breeding center of Wenshu County, Aksu Prefecture, Xinjiang Province. They belonged from the Aerken group (Numbers(n) = 8), Samusak group (n = 8), Tuniazi group (n = 8), and Yiming group (n = 8). A total of 32 1-year-old female Tibetan cashmere goats were selected from the original breed of cashmere goats in Ngari Prefecture (Ritu County, n = 9; Gaize County, n = 11) and Nagqu Prefecture (Nima County, n = 12)(Fig. 1A). Before the experiment, all cashmere goats were healthy and raised by grazing.

### Sample collection

Cashmere samples were collected from Jiangnan and Tibetan cashmere goats from the 10 cm posterior margin of the scapula above the body’s left midline. Cashmere is naturally dried after washing according to a conventional process. A fiber diameter optical analyzer (OFDA2000) was used to determine the cashmere mean fiber diameter (MFD)(Table S5), fiber diameter standard deviation (FDSD), and coefficient of variation of fiber diameter (CVFD) under a constant temperature of 20℃ ± 2℃ and humidity of 65% ± 4%.

Together with the cashmere sample, we collected 5 mL of blood in an anticoagulation tube and stored it in a refrigerator at − 20℃. The cashmere mean fiber diametkit (TIANGENG, USA) was used to extract DNA from cashmere goats. DNA quality and concentration were determined using 1.0% agarose gel electrophoresis and the Qubit 2.0 (Thermo, USA). The results of agarose gel electrophoresis of 64 DNA samples are showed in Figure S5.

### Whole-genome resequencing

A total of 64 DNA samples, including 32 Jiangnan cashmere goats and 32 Tibetan cashmere goats, were analyzed. Sequencing library construction was performed as follows: 1 µg of genomic DNA was randomly cut with Covaris; then, the fragments were screened with Agent AMPure XP-Medium Kit with an average size of 200–400 bp. The 64 single strand circle DNAs were formatted as final qualified libraries, which were sequenced at 10× average depth by BGISEQ-500.

### Variant discovery and genotyping

High-quality sequencing data of 64 cashmere goats were mapped to the goat reference genome (ARS1.2, GCA_001704415.2) by using BWA software (Parameter: mem-t 4-M) [51]; the mapped results were deduplicated using the SAMTOOLS software (Parameter: rmdup) [52]. GATK software [53] was used to detect SNPs in multiple samples; high-quality SNPs were obtained by filtering and screening under the following conditions:

1) Supporting number (depth of coverage) of SNPs ≥ 2.

2) The proportion of MIS (missing) < 10%.

3) Minimum allele frequency > 5%.

### Principal component and phylogenetic analysis

Principal component analysis (PCA) was based on all SNPs using GCTA software [54]. Subsequently, the Treebest-1.9.2 software (http://treesoft.sourceforge.net/index.shtml) was used to calculate the distance matrix also based on the SNPs of individuals. The distance between two individuals i and j was calculated by the following formula:

$${\text{D}\text{i}\text{s}\text{t}\text{a}\text{n}\text{c}\text{e}}_{\text{i}\text{j}}=\frac{1}{L}\sum _{l=1}^{L}{d}_{ij}^{\left(1\right)}$$

L in the formula is the length of the region of high quality SNPs, assuming that the allele at position 1 is A/C, then:

1. If the genotypes of both individuals are AA:${d}_{ij}^{\left(1\right)}=0$
2. If the genotypes of two individuals are AA and AC respectively:${d}_{ij}^{\left(1\right)}=0.5$
3. If the genotypes of both individuals are AC:${d}_{ij}^{\left(1\right)}=0.5$
4. If the genotypes of two individuals are AA and CC respectively:${d}_{ij}^{\left(1\right)}=1$

And on this basis a phylogenetic tree was constructed using the neighbor-joining method. Bootstrap values were obtained after up to 1000 calculations.

### Structural analysis

We estimated the ancestry of each individual using the genome-wide unlinked SNP dataset and the model-based assignment software program ADMIXTURE [55] to quantify genome-wide admixture between Jiangnan and Tibetan cashmere goats. ADMIXTURE was run for each possible group number (K = 2, 3, and 4) with 200 bootstrap replicates to estimate the parameter standard errors used to determine the optimal group number (K).

### Linkage disequilibrium (LD) analysis

We separated the 64 samples by breed and cashmere fineness, and used Haploview software [56] to calculate the r^2^ for each of the four populations (Fine cashmere (Fe) group: JF and TF; Coarse cashmere (Ce) group: JC and TC). It is generally accepted that there is a linkage between SNPs in the range of r^2^≥r^2^_MAX_/2. An r^2^ value of 0.175 was used for subsequent analysis. When the r^2^ value is 0.175, the distance is 20 Kb.

### Genome scanning for potential selected genes

To identify the potential selected genes (PSGs) that have undergone environmental adaptive selection and investigate the differences between cashmere goat breeds, we measured the allele frequency (FST) and the genetic diversity (Pi (π)) ratio was compared using a sliding window (5 kb window) by using VCFtools. Meanwhile, 5% of windows with the highest FST and Pi (π) ratio were considered candidate divergent windows, thereby becoming the potential selected region. The Pi (π) ratio between Jiangnan and Tibetan cashmere goat or between Tibetan and Jiangnan cashmere goatswas calculated as log_2_(Pi ratio(PiJ/PiT)) or log_2_(Pi ratio(PiT/PiJ)), reflecting the loss of nucleotide diversity in Jiangnan cashmere goat relative to Tibetan cashmere goat or Tibetan cashmere goat relative to Jiangnan cashmere goat.

### Genome-wide association analysis

Genome-wide association analysis (GWAS) of cashmere fineness traits (including MFD, FDSD and CVFD) in the goat population (including 32 Jiangnan and 32 Tibetan cashmere goats) was performed using GEMMA [57], using the significance (*P*-value) of the association to screen out potential candidate SNPs. In this study SNPs with -log_10_(P)>4 were considered significant SNPs. During GWAS, individual relationship and population stratification were the main factors causing false-positive associations. Therefore, a mixed linear model was used for GWAS, using a population genetic structure as a fixed effect and individual relationships as a random effect to correct for the effects of population structure and individual relationships:

$$y=X\alpha +Z\beta +W\mu +e$$

where y is the phenotypic trait, X is the indicator matrix of the fixed effect, α is the estimated parameter of the fixed effect; Z is the indicator matrix of the SNP, β is the effect of the SNP; W is the only matrix of the random effect, µ is the predicted random individual, and e is the random residual, obeying e~(0, δe2).

### Transcriptomic analysis

The 16 goat transcriptomes were obtained from the NCBI of SRA (https://www.ncbi.nlm.nih.gov/sra/?term=), including eight mature female Jiangnan (BioProject: PRJNA778726) and eight mature female Tibetan (BioProject: PRJNA643003) cashmere goat skin tissue transcriptomes. SRA Toolkit was used to download these data. The clean reads of the 16 transcriptomes were mapped to the goat reference genome (ARS1.2, GCA_001704415.2) using HISAT 2.0 software [58]; the mapping rates of all samples were > 95%. Then, transcript assembly and quantification were performed using StringTie software [59], and fragments per kilobase million (FPKM) was used as a measure of transcript or gene expression levels. The genome-wide gene expression pattern analysis of the 16 samples was performed using WGCNA software [60]. Finally, differentially expressed gene (DEG) analysis was performed using DESeq 2.0 [61].

### Functional enrichment analysis of candidate genes

GO and KEGG annotation of candidate genes was performed using the online tool DAVID (https://david.ncifcrf.gov/) with default parameters. The GO annotation is divided into three levels: Biological process, Cellular component, and Molecular function.

## Electronic supplementary material

Below is the link to the electronic supplementary material.

Supplementary Material 1

Supplementary Material 2

Supplementary Material 3

Supplementary Material 4

Supplementary Material 5

Supplementary Material 6

Supplementary Material 7

Supplementary Material 8

Supplementary Material 9

Supplementary Material 10

## Data Availability

All the genome sequencing data have been deposited in the SRA of NCBI. PRJNA897713: Genome resequencing data of Tibetan cashmere goats. PRJNA898437: Genome resequencing data of Jiangnan cashmere goats.
