# Supplementary figures and images for: Drivers of plateau adaptability in cashmere goats revealed by genomic and transcriptomic analyses
Source: BMC Genomics. 2023 Aug 1;24:428. doi: 10.1186/s12864-023-09333-1 (PMC10391913; doi:10.1186/s12864-023-09333-1)

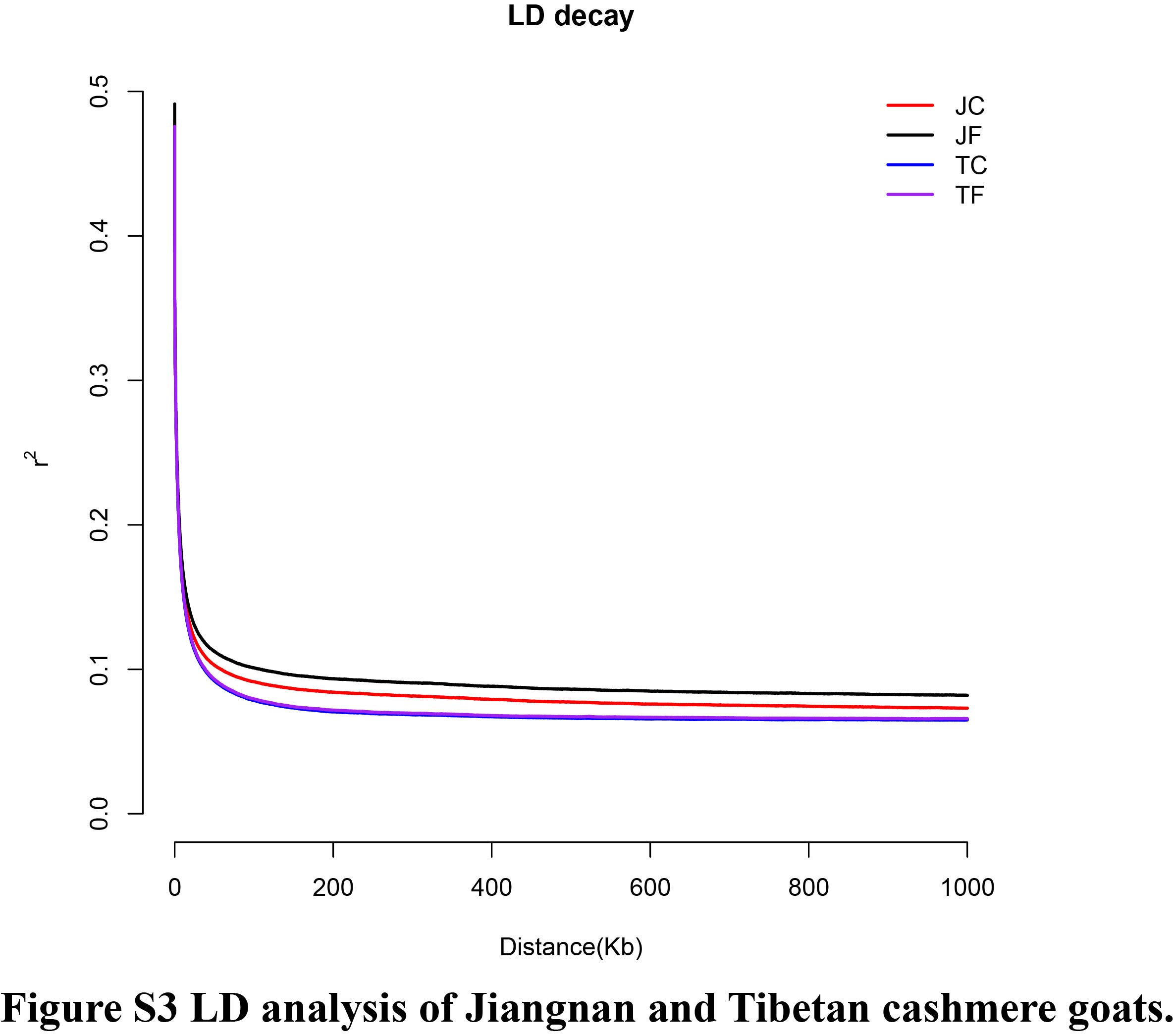

Supplement: Supplementary file 6 — Supplementary Material 6 [file 12864_2023_9333_MOESM6_ESM.jpg]

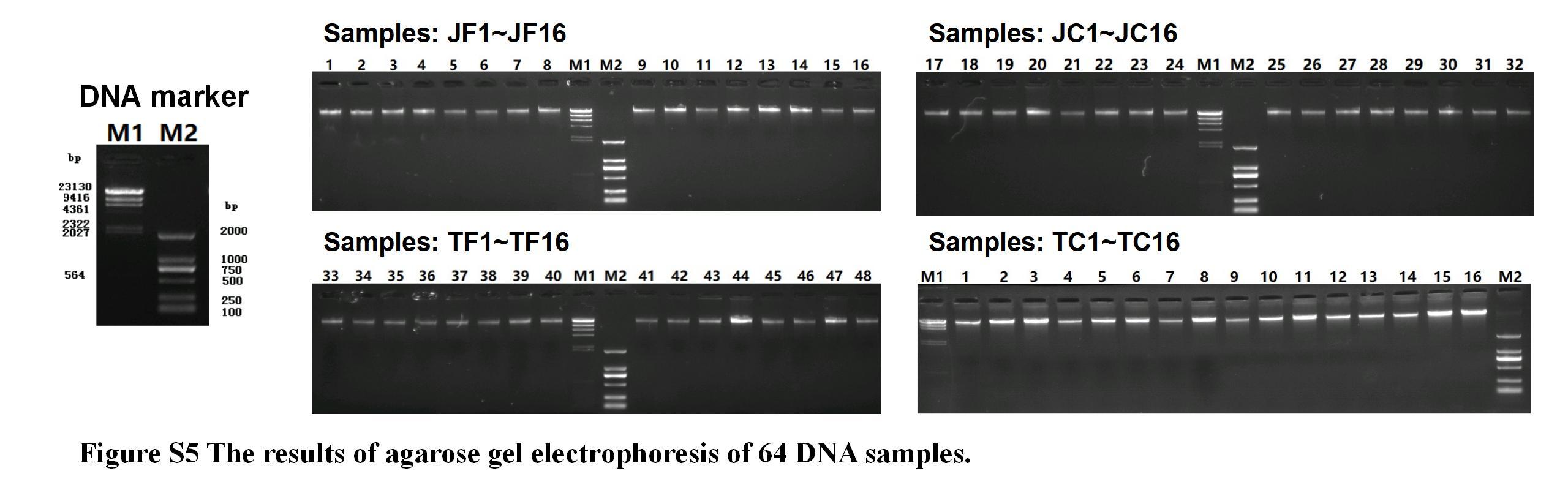

Supplement: Supplementary file 9 — Supplementary Material 9 [file 12864_2023_9333_MOESM9_ESM.jpg]

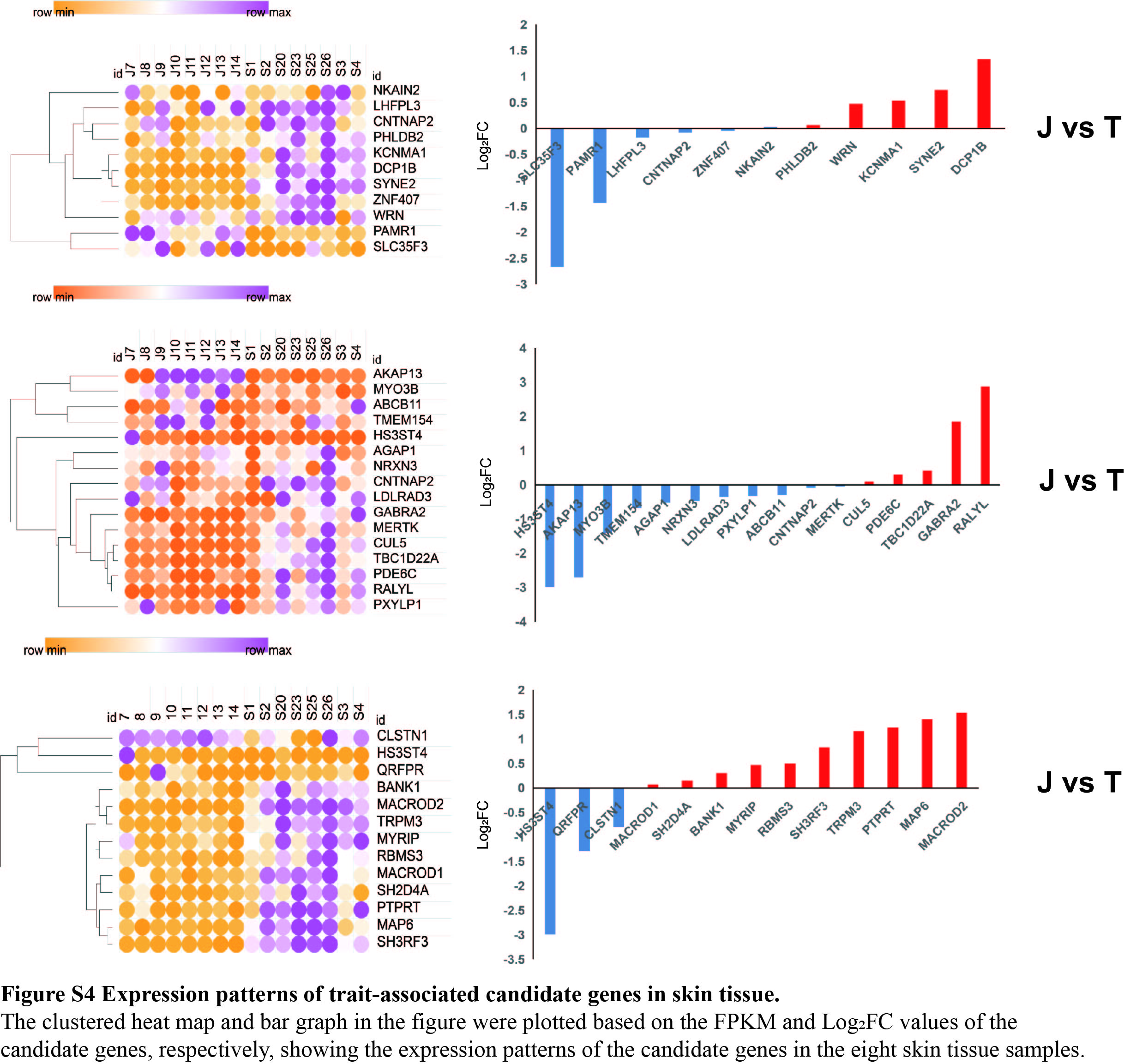

Supplement: Supplementary file 10 — Supplementary Material 10 [file 12864_2023_9333_MOESM10_ESM.jpg]
